# Supplementary material for: Molecular Detection of Metronidazole and Tetracycline Resistance Genes in Helicobacter pylori-Like Positive Gastric Samples from Pigs
Source: Antibiotics (Basel). 2023 May 13;12(5):906. doi: 10.3390/antibiotics12050906 (PMC10215371; doi:10.3390/antibiotics12050906)
Supplement: Supplementary file 1 [file antibiotics-12-00906-s001.zip › antibiotics-2390066-supplementary.pdf]

Supplementary Materials

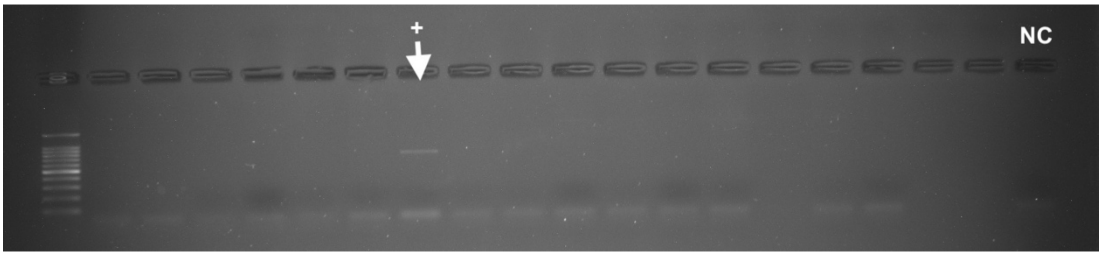

Figure S1. *frxA* PCR products electrophoresis gel; +: *frxA* positive sample; NC - Negative control

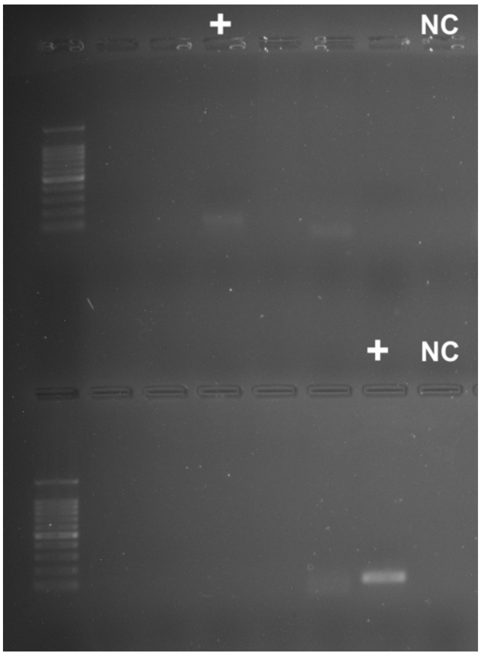

Figure S2. *16s rRNA* PCR products electrophoresis gel; +: *16s rRNA* positive samples confirmed by sequencing ; NC - Negative control

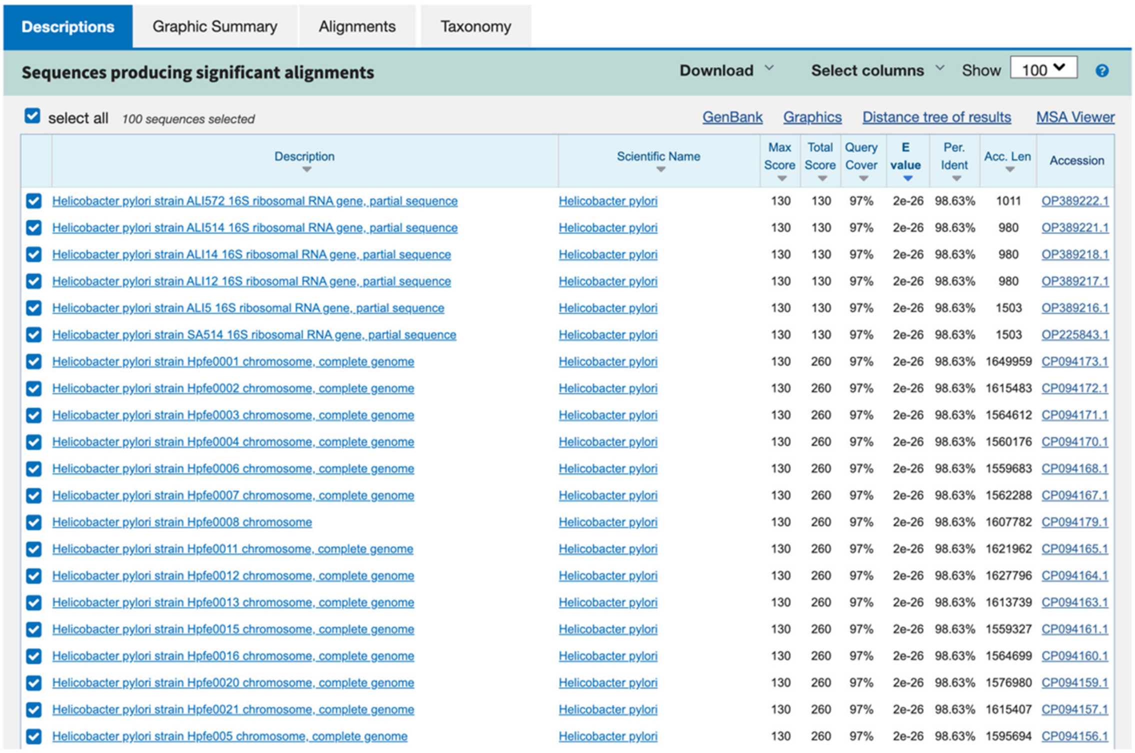

Descriptions | Graphic Summary | Alignments | Taxonomy

Sequences producing significant alignments Download Select columns Show 100

select all *100 sequences selected* GenBank Graphics Distance tree of results MSA Viewer

Description	Scientific Name	Max Score	Total Score	Query Cover	E value	Per. Ident	Acc. Len	Accession
Helicobacter pylori strain ALI572 16S ribosomal RNA gene, partial sequence	Helicobacter pylori	130	130	97%	2e-26	98.63%	1011	OP389222.1
Helicobacter pylori strain ALI514 16S ribosomal RNA gene, partial sequence	Helicobacter pylori	130	130	97%	2e-26	98.63%	980	OP389221.1
Helicobacter pylori strain ALI14 16S ribosomal RNA gene, partial sequence	Helicobacter pylori	130	130	97%	2e-26	98.63%	980	OP389218.1
Helicobacter pylori strain ALI12 16S ribosomal RNA gene, partial sequence	Helicobacter pylori	130	130	97%	2e-26	98.63%	980	OP389217.1
Helicobacter pylori strain ALI5 16S ribosomal RNA gene, partial sequence	Helicobacter pylori	130	130	97%	2e-26	98.63%	1503	OP389216.1
Helicobacter pylori strain SA514 16S ribosomal RNA gene, partial sequence	Helicobacter pylori	130	130	97%	2e-26	98.63%	1503	OP225843.1
Helicobacter pylori strain Hpfe0001 chromosome, complete genome	Helicobacter pylori	130	260	97%	2e-26	98.63%	1649959	CP094173.1
Helicobacter pylori strain Hpfe0002 chromosome, complete genome	Helicobacter pylori	130	260	97%	2e-26	98.63%	1615483	CP094172.1
Helicobacter pylori strain Hpfe0003 chromosome, complete genome	Helicobacter pylori	130	260	97%	2e-26	98.63%	1564612	CP094171.1
Helicobacter pylori strain Hpfe0004 chromosome, complete genome	Helicobacter pylori	130	260	97%	2e-26	98.63%	1560176	CP094170.1
Helicobacter pylori strain Hpfe0006 chromosome, complete genome	Helicobacter pylori	130	260	97%	2e-26	98.63%	1559683	CP094168.1
Helicobacter pylori strain Hpfe0007 chromosome, complete genome	Helicobacter pylori	130	260	97%	2e-26	98.63%	1562288	CP094167.1
Helicobacter pylori strain Hpfe0008 chromosome	Helicobacter pylori	130	260	97%	2e-26	98.63%	1607782	CP094179.1
Helicobacter pylori strain Hpfe0011 chromosome, complete genome	Helicobacter pylori	130	260	97%	2e-26	98.63%	1621962	CP094165.1
Helicobacter pylori strain Hpfe0012 chromosome, complete genome	Helicobacter pylori	130	260	97%	2e-26	98.63%	1627796	CP094164.1
Helicobacter pylori strain Hpfe0013 chromosome, complete genome	Helicobacter pylori	130	260	97%	2e-26	98.63%	1613739	CP094163.1
Helicobacter pylori strain Hpfe0015 chromosome, complete genome	Helicobacter pylori	130	260	97%	2e-26	98.63%	1559327	CP094161.1
Helicobacter pylori strain Hpfe0016 chromosome, complete genome	Helicobacter pylori	130	260	97%	2e-26	98.63%	1564699	CP094160.1
Helicobacter pylori strain Hpfe0020 chromosome, complete genome	Helicobacter pylori	130	260	97%	2e-26	98.63%	1576980	CP094159.1
Helicobacter pylori strain Hpfe0021 chromosome, complete genome	Helicobacter pylori	130	260	97%	2e-26	98.63%	1615407	CP094157.1
Helicobacter pylori strain Hpfe005 chromosome, complete genome	Helicobacter pylori	130	260	97%	2e-26	98.63%	1595694	CP094156.1

Figure S3. Results of the basic alignment research tool for sequenced sample 1, *16s rRNA* gene.

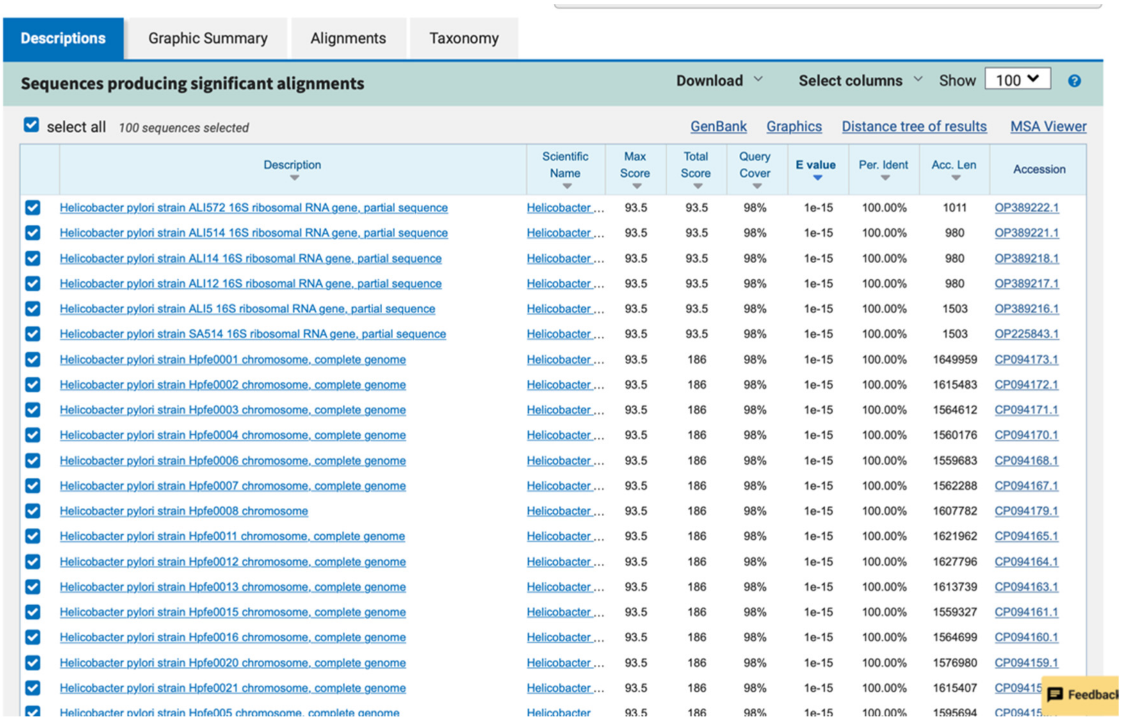

Descriptions | Graphic Summary | Alignments | Taxonomy

Sequences producing significant alignments Download Select columns Show 100

select all *100 sequences selected* GenBank Graphics Distance tree of results MSA Viewer

Description	Scientific Name	Max Score	Total Score	Query Cover	E value	Per. Ident	Acc. Len	Accession
Helicobacter pylori strain ALI572 16S ribosomal RNA gene, partial sequence	Helicobacter ...	93.5	93.5	98%	1e-15	100.00%	1011	OP389222.1
Helicobacter pylori strain ALI514 16S ribosomal RNA gene, partial sequence	Helicobacter ...	93.5	93.5	98%	1e-15	100.00%	980	OP389221.1
Helicobacter pylori strain ALI14 16S ribosomal RNA gene, partial sequence	Helicobacter ...	93.5	93.5	98%	1e-15	100.00%	980	OP389218.1
Helicobacter pylori strain ALI12 16S ribosomal RNA gene, partial sequence	Helicobacter ...	93.5	93.5	98%	1e-15	100.00%	980	OP389217.1
Helicobacter pylori strain ALI5 16S ribosomal RNA gene, partial sequence	Helicobacter ...	93.5	93.5	98%	1e-15	100.00%	1503	OP389216.1
Helicobacter pylori strain SA514 16S ribosomal RNA gene, partial sequence	Helicobacter ...	93.5	93.5	98%	1e-15	100.00%	1503	OP225843.1
Helicobacter pylori strain Hpfe0001 chromosome, complete genome	Helicobacter ...	93.5	186	98%	1e-15	100.00%	1649959	CP094173.1
Helicobacter pylori strain Hpfe0002 chromosome, complete genome	Helicobacter ...	93.5	186	98%	1e-15	100.00%	1615483	CP094172.1
Helicobacter pylori strain Hpfe0003 chromosome, complete genome	Helicobacter ...	93.5	186	98%	1e-15	100.00%	1564612	CP094171.1
Helicobacter pylori strain Hpfe0004 chromosome, complete genome	Helicobacter ...	93.5	186	98%	1e-15	100.00%	1560176	CP094170.1
Helicobacter pylori strain Hpfe0006 chromosome, complete genome	Helicobacter ...	93.5	186	98%	1e-15	100.00%	1559683	CP094168.1
Helicobacter pylori strain Hpfe0007 chromosome, complete genome	Helicobacter ...	93.5	186	98%	1e-15	100.00%	1562288	CP094167.1
Helicobacter pylori strain Hpfe0008 chromosome	Helicobacter ...	93.5	186	98%	1e-15	100.00%	1607782	CP094179.1
Helicobacter pylori strain Hpfe0011 chromosome, complete genome	Helicobacter ...	93.5	186	98%	1e-15	100.00%	1621962	CP094165.1
Helicobacter pylori strain Hpfe0012 chromosome, complete genome	Helicobacter ...	93.5	186	98%	1e-15	100.00%	1627796	CP094164.1
Helicobacter pylori strain Hpfe0013 chromosome, complete genome	Helicobacter ...	93.5	186	98%	1e-15	100.00%	1613739	CP094163.1
Helicobacter pylori strain Hpfe0015 chromosome, complete genome	Helicobacter ...	93.5	186	98%	1e-15	100.00%	1559327	CP094161.1
Helicobacter pylori strain Hpfe0016 chromosome, complete genome	Helicobacter ...	93.5	186	98%	1e-15	100.00%	1564699	CP094160.1
Helicobacter pylori strain Hpfe0020 chromosome, complete genome	Helicobacter ...	93.5	186	98%	1e-15	100.00%	1576980	CP094159.1
Helicobacter pylori strain Hpfe0021 chromosome, complete genome	Helicobacter ...	93.5	186	98%	1e-15	100.00%	1615407	CP09415[illegible]
Helicobacter pylori strain Hpfe005 chromosome, complete genome	Helicobacter	93.5	186	98%	1e-15	100.00%	1595694	CP09415[illegible]

Feedback

Figure S4. Results of the basic alignment research tool for sequenced sample 2, *16s rRNA* gene.

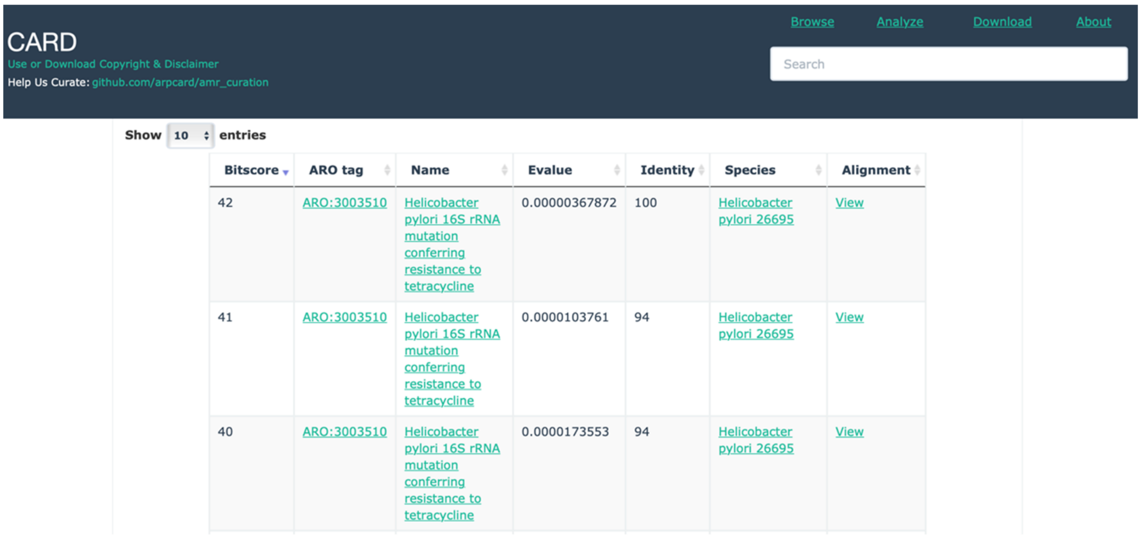

Bitscore	ARO tag	Name	Evalue	Identity	Species	Alignment
42	ARO:3003510	Helicobacter pylori 16S rRNA mutation conferring resistance to tetracycline	0.00000367872	100	Helicobacter pylori 26695	View
41	ARO:3003510	Helicobacter pylori 16S rRNA mutation conferring resistance to tetracycline	0.0000103761	94	Helicobacter pylori 26695	View
40	ARO:3003510	Helicobacter pylori 16S rRNA mutation conferring resistance to tetracycline	0.0000173553	94	Helicobacter pylori 26695	View

Figure S5. CARD analysis of PCR-positive samples for *16s rRNA* gene, sample 1.

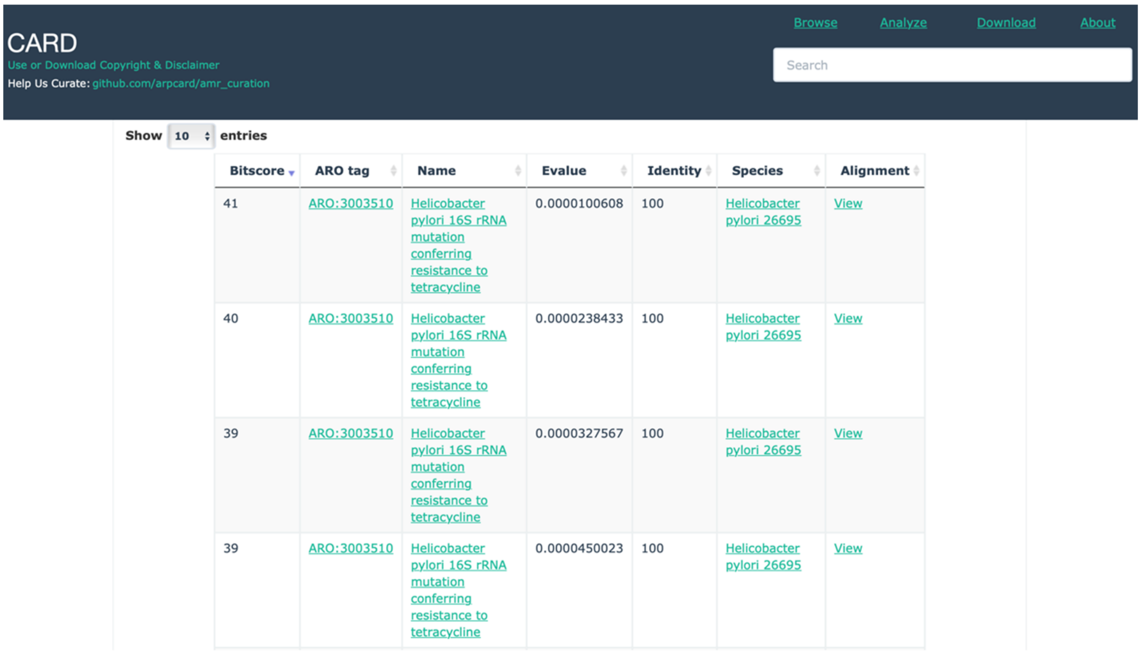

Bitscore	ARO tag	Name	Evalue	Identity	Species	Alignment
41	ARO:3003510	Helicobacter pylori 16S rRNA mutation conferring resistance to tetracycline	0.0000100608	100	Helicobacter pylori 26695	View
40	ARO:3003510	Helicobacter pylori 16S rRNA mutation conferring resistance to tetracycline	0.0000238433	100	Helicobacter pylori 26695	View
39	ARO:3003510	Helicobacter pylori 16S rRNA mutation conferring resistance to tetracycline	0.0000327567	100	Helicobacter pylori 26695	View
39	ARO:3003510	Helicobacter pylori 16S rRNA mutation conferring resistance to tetracycline	0.0000450023	100	Helicobacter pylori 26695	View

Figure S6. CARD analysis of PCR-positive samples for *16s rRNA* gene, sample 2.

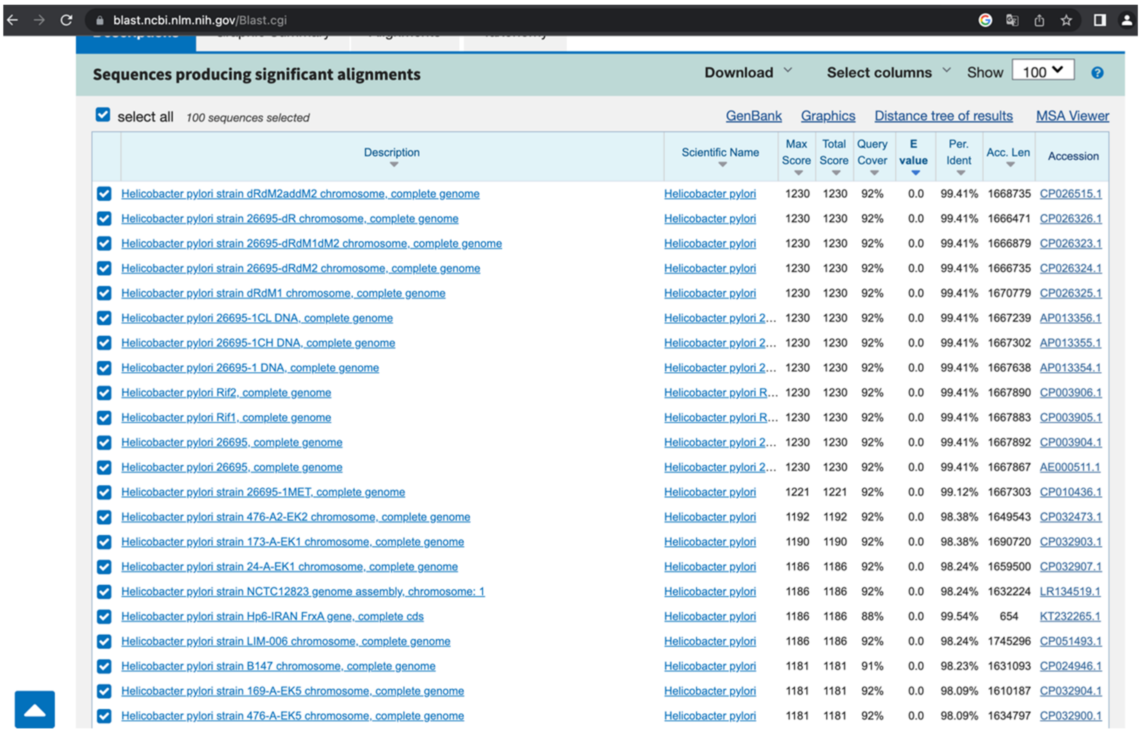

Figure S7. Results of the basic alignment research tool for sequenced sample, *frxA*

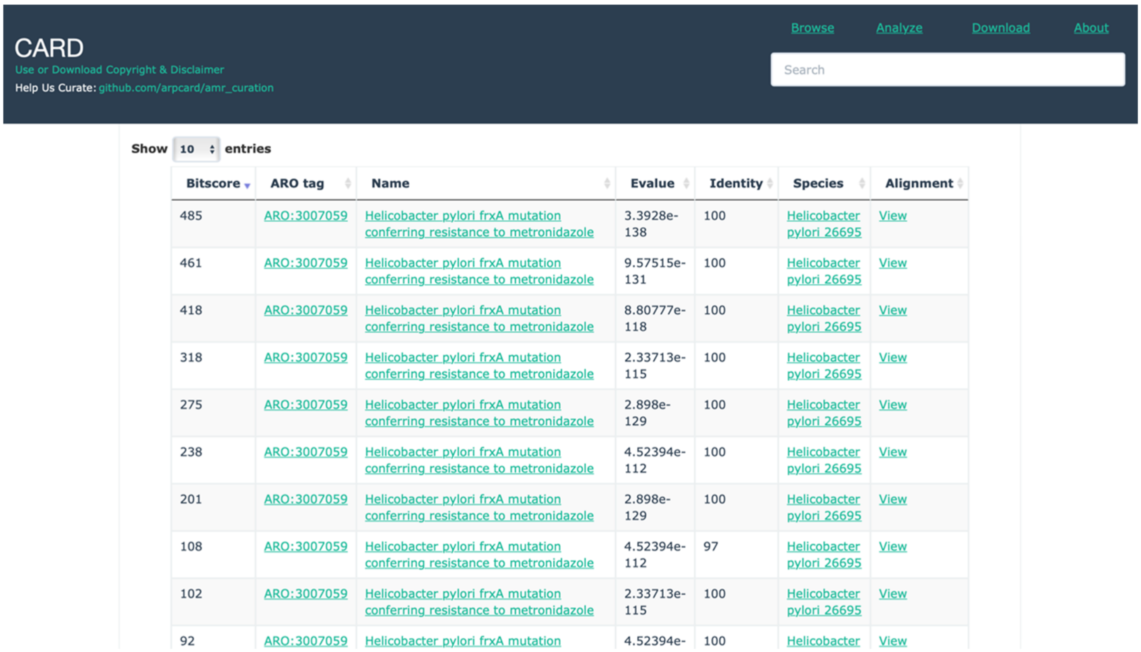

Figure S8. CARD analysis of the *frxA* mutation gene positive sample sequence.

Results (all Loose hits shown)

Search:

RGI Criteria	ARO Term	SNP	Detection Criteria	AMR Gene Family	Drug Class	Resistance Mechanism	% Identity of Matching Region	% Length of Reference Sequence
Strict	Helicobacter pylori frxA mutation conferring resistance to metronidazole	Y62D	protein variant model	Antibiotic resistant Helicobacter pylori nitroreductase	nitroimidazole antibiotic	antibiotic target alteration	99.07	108.29

Previous 1 Next

Figure S9. Resistance gene identifier analysis of the *frxA* mutation gene positive sample sequence

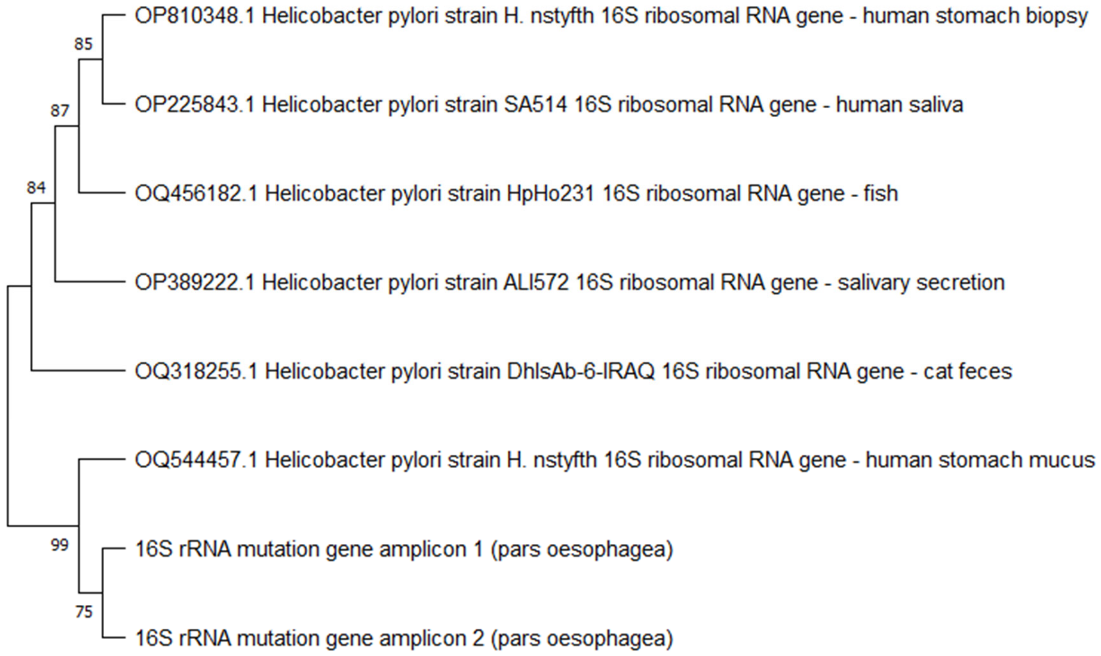

Figure S10. Phylogenetic tree based on comparison of *16S rRNA* gene sequences.
The evolutionary history was inferred using the Neighbor-Joining method [1]. The bootstrap consensus tree inferred from 1000 replicates [2] is taken to represent the evolutionary history of the taxa analyzed [2]. Branches corresponding to partitions reproduced in less than 50% bootstrap replicates are collapsed. The percentage of replicate trees in which the associated taxa clustered together in the bootstrap test (1000 replicates) are shown next to the branches [2]. The evolutionary distances were computed using the Maximum Composite Likelihood method [3] and are in the units of the number of base substitutions per site. This analysis involved 8 nucleotide sequences which were aligned using ClustalW. All ambiguous positions were removed for each sequence pair (pairwise deletion option). There were a total of 1503 positions in the final dataset. Evolutionary analyses were conducted in MEGA11 [4].
Following the hyphen, the sample type from which the corresponding *H. pylori* strain was isolated is shown.

1. Saitou, N.; Nei, M. The neighbor-joining method: A new method for reconstructing phylogenetic trees. *Molecular Biology and Evolution* **1987**, *4*, 406-425.
2. Felsenstein, J. Confidence limits on phylogenies: An approach using the bootstrap. *Evolution* **1985**, *39*, 783-791.
3. Tamura, K.; Nei, M.; Kumar, S. Prospects for inferring very large phylogenies by using the neighbor-joining method. *Proceedings of the National Academy of Sciences* (*USA*) **2004**, *101*, 11030-11035.
4. Tamura, K.; Stecher, G.; Kumar, S. MEGA 11: Molecular Evolutionary Genetics Analysis Version 11. *Molecular Biology and Evolution* **2021**, https://doi.org/10.1093/molbev/msab120.

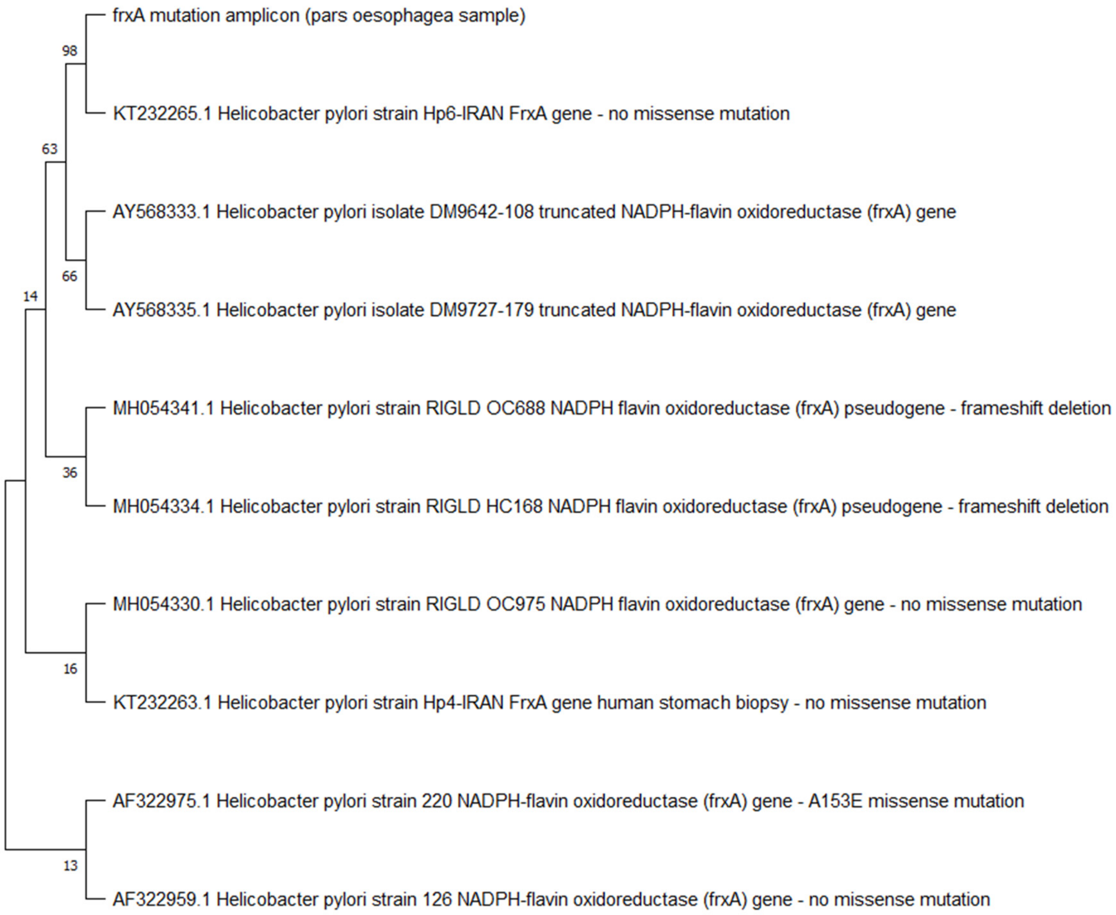

Figure S11. Phylogenetic tree based on comparison of *frxA* gene sequences.
The evolutionary history was inferred using the Neighbor-Joining method [1]. The bootstrap consensus tree inferred from 1000 replicates [2] is taken to represent the evolutionary history of the taxa analyzed [2]. Branches corresponding to partitions reproduced in less than 50% bootstrap replicates are collapsed. The percentage of replicate trees in which the associated taxa clustered together in the bootstrap test (1000 replicates) are shown next to the branches [2]. The evolutionary distances were computed using the Maximum Composite Likelihood method [3] and are in the units of the number of base substitutions per site. This analysis involved 10 nucleotide sequences which were aligned using ClustalW. All ambiguous positions were removed for each sequence pair (pairwise deletion option). There were a total of 735 positions in the final dataset. Evolutionary analyses were conducted in MEGA11 [4].
All reference strain sequences included, originated from *H. pylori* strains isolated from human gastric biopsy samples which showed metronidazole resistance with minimum inhibitory concentrations ranging from 16 to 256 mg/l. Following the hyphen, it is indicated what mutations were present in the reference strain sequences.

1. Saitou, N.; Nei, M. The neighbor-joining method: A new method for reconstructing phylogenetic trees. *Molecular Biology and Evolution* **1987**, *4*, 406-425.
2. Felsenstein, J. Confidence limits on phylogenies: An approach using the bootstrap. *Evolution* **1985**, *39*, 783-791.
3. Tamura, K.; Nei, M.; Kumar, S. Prospects for inferring very large phylogenies by using the neighbor-joining method. *Proceedings of the National Academy of Sciences* (*USA*) **2004**, *101*, 11030-11035.
4. Tamura, K.; Stecher, G.; Kumar, S. MEGA 11: Molecular Evolutionary Genetics Analysis Version 11. *Molecular Biology and Evolution* **2021**, https://doi.org/10.1093/molbev/msab120.

Table S1. Primer sequences used for detection of *Helicobacter pylori*(-like organisms) and thermocycling conditions in previous research

Primer	Sequence	Target gene	Amplicon size	Thermal cycle conditions			*Helicobacter* strain used as positive control	Result of samples included in current study
				Nr. Cycles	Temp. (ºC)	Time		
BFHpyl_F1	AAA gAg CgT ggT TTT CAT ggC g	*ureAB*	217 bp	45	94<br>59<br>72	30 sec<br>30 sec<br>1 min	26695	Positive
BFHpyl_R1	ggg TTT TAC CgC CAC CgA ATT TAA							
Hpy3F	TTATCGGTAAAGACACC AGAAA	*glmM*	144	45	94<br>54<br>72	30 sec<br>30 sec<br>30 sec	SS1	Negative
Hpy3R	ATCACAGCGCATGTCTTC							

From: Cortez Nunes, F.; Letra Mateus, T.; Taillieu, E.; Teixeira, S.; Carolino, N.; Rema, A.; De Bruyckere, S.; Gärtner, F.; Haesebrouck, F.; Amorim, I. Molecular detection of *Helicobacter* spp. and *Fusobacterium gastrosuis* in pigs and wild boars and its association with gastric histopathological alterations. *Vet Res* **2022**, *53*, 78, doi:10.1186/s13567-022-01101-5.
